# Outcomes and Characteristics of Patients Undergoing Percutaneous Angioplasty Followed by Below-Knee or Above-Knee Amputation for Peripheral Artery Disease

**DOI:** 10.1371/journal.pone.0111130

**Published:** 2014-10-29

**Authors:** Chun-Tai Mao, Ming-Lung Tsai, Chao-Yung Wang, Ming-Shien Wen, I-Chang Hsieh, Ming-Jui Hung, Chao-Hung Wang, Chun-Chi Chen, Tien-Hsing Chen

**Affiliations:** 1 Heart Failure Center, Division of Cardiology, Department of Internal Medicine, Chang Gung Memorial Hospital, Keelung, Taiwan; 2 Division of Cardiology, Department of Internal Medicine, Chang Gung Memorial Hospital, Taipei, Taiwan; 3 Department of cardiology, Chang Gung Memorial Hospital, Xiamen, China; 4 College of Medicine, Chang Gung University, Taoyuan, Taiwan; Osaka University Graduate School of Medicine, Japan

## Abstract

**Objective:**

Little is known about long-term outcomes among patients who receive percutaneous angioplasty (PTA) for peripheral artery disease (PAD) then undergo below-knee or above-knee amputations. We sought to determine clinical outcomes associated with below-knee or above-knee amputation, along with possible explanatory factors and treatment strategies.

**Methods:**

Using data from Taiwan’s National Health Insurance Research Database from 1997 to 2010, 7,568 adult patients were divided into three groups: lower extremity preserved (LE), below-knee amputation (BK) and above-knee amputation (AK). We assessed outcomes including major adverse cardiovascular events (MACE) and associated risk factors.

**Results:**

Overall MACE was significantly higher in the AK group compared to the LE and BK groups, over a mean follow-up of 2.45 years (hazard ratio [HR]: 1.81; 95% confidence interval [CI]: 1.50–2.18 for AK vs. LE; HR: 1.67; 95% CI: 1.36–2.06 for AK vs. BK). However MACE were similar for the BK and LE groups (HR: 1.08; 95% CI: 0.98–1.20). Overall mortality was highest in the AK group (HR: 1.65, 95% CI: 1.34–2.04 for AK vs. BK). As for patient characteristics, atrial fibrillation was more prevalent in the AK group than in the BK group (17% vs. 7%). Independent risk factors associated with death after above- or below-knee amputation included advanced age, heart failure, dialysis, male gender and high patient volume.

**Conclusion:**

The MACE rate was highest in the AK group, whereas the LE and BK groups were similar in this regard. Furthermore, overall mortality increased with larger area of amputation.

## Introduction

As societies age, the incidence of peripheral artery disease (PAD) has been increasing, along with the increase in patients with risk factors for atherosclerosis, especially diabetes mellitus [1], [2]. Patients with PAD have a high probability of suffering from critical limb ischemia (CLI) which may require major lower extremity amputation, including above-knee (AK) or below-knee (BK) amputations [2]. Prior studies have revealed that such patients face high morbidity and health costs [3]–[7]. Furthermore, major lower extremity amputations may contribute to high mortality [8]–[10]. Therefore revascularization, including bypass surgery and percutaneous angioplasty (PTA), should be considered before amputation, according to the American College of Cardiology/American Heart Association guideline recommendations [11]. In the real world, however, bypass surgery is rarely applied to patients with CLI, because such patients commonly have higher incidence of comorbidities and advanced age, making PTA the preferred treatment [2], [12]. After PTA, patients may be able to keep their lower extremity, or they may receive BK or AK amputation. Little is known about long-term and short-term outcomes for these three groups of patients–those receiving only PTA, and those receiving PTA followed by BK, or receiving PTA followed by AK amputation. We evaluated the outcomes in terms of myocardial infarction (MI), stroke, and all causes of mortality. The clinical prognoses of these patients vary based on a number of factors, some of which are patient specific, but no comprehensive report thus far has evaluated the effect of such factors on cardiovascular outcomes.

To evaluate outcomes among PTA patients who kept their lower extremity or received AK or BK amputation, we analyzed data from Taiwan’s National Healthcare Insurance (NHI) Program. Our specific aims were to (1) determine rates of death, MI, and stroke among patients who kept their leg or received AK or BK amputation following PTA; (2) determine factors associated with clinical outcomes in the three groups; and (3) determine the major causes of death.

## Methods

### 1. Data source

We conducted a nationwide population-based cohort study using Taiwan’s National Health Insurance Research Database (NHIRD), which consists of standard computerized claims documents submitted by medical institutions seeking reimbursement through the NHI Program. PTA is an option covered by the NHI, and patients with PAD who received PTA are recorded in the NHIRD.

The NHI program covers medical needs for 99.19% of the population in Taiwan, more than 25 million people. The accuracy and validation of NHIRD data is based on regular auditing of claims by the NHI Bureau [13]–[16]. False reimbursement claims result in substantial penalties. Minor infractions involve fines of 100 times the amount of the false claim, while serious infractions may result in revoking physicians’ licenses or criminal charges.

This study was approved by the Ethics Institutional Review Board of Chang Gung Memorial Hospital. The information and records of patients were anonymized and de-identified prior to analysis.

### 2. Study Cohorts and Follow-up

**Figure 1:**
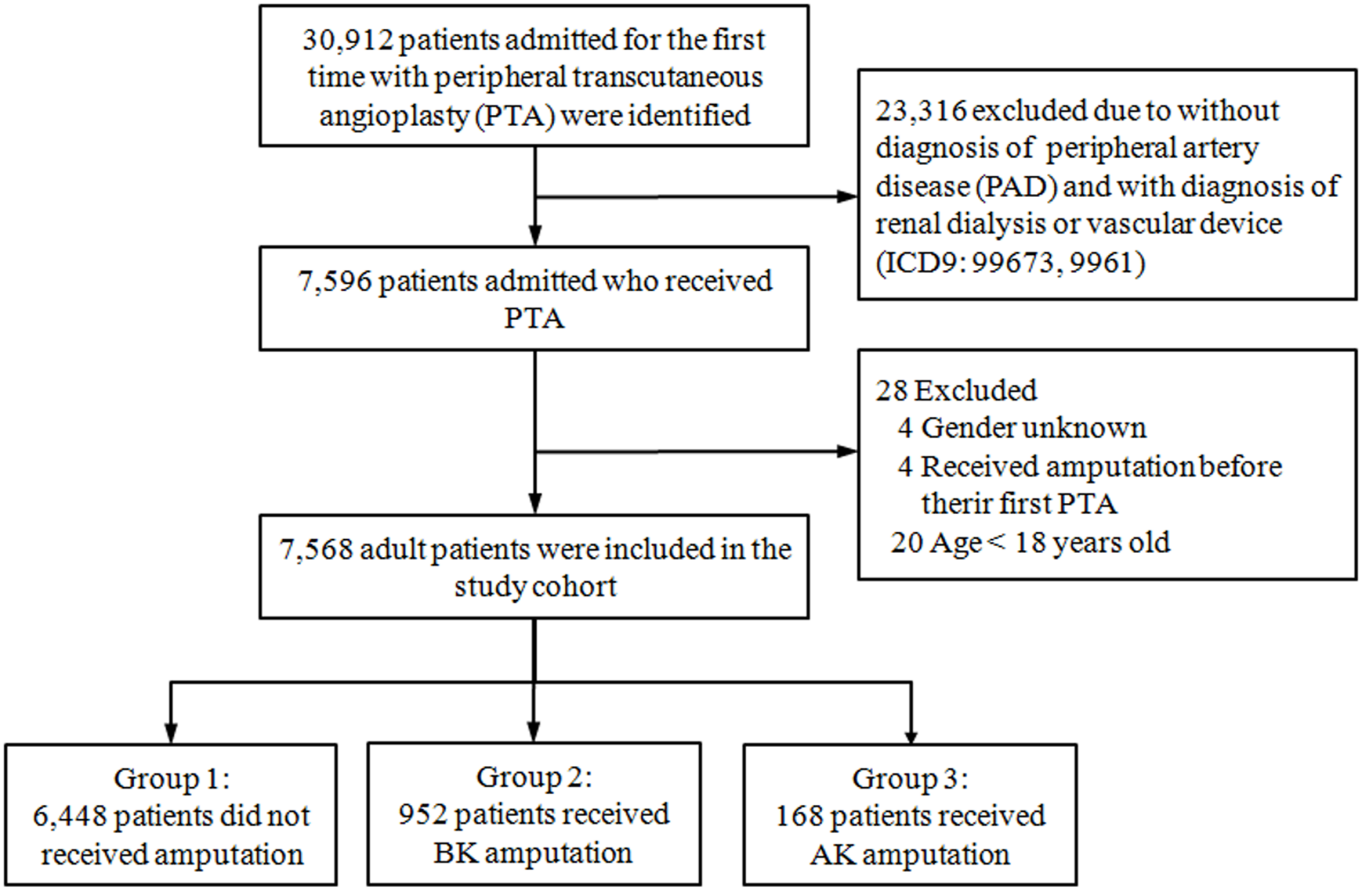
Enrollment flow chart.

We identified all patients in the NHIRD who received PTA between January 1, 1997 and December 31, 2010 and considered first time admission for PTA as the index hospitalization. The study excluded patients with arterio-venous shunt dysfunction in end stage renal disease who may have received PTA for this clinical event. After we selected this initial cohort of PAD patients receiving PTA, we divided them into three groups according to the results of their index hospitalization. The lower extremity preserved (LE) group consisted of patients whose lower extremity were preserved after PTA. The BK group included patients who received BK amputation following PTA. The AK group included patients receiving AK amputation after PTA (Fig. 1). Follow-up was from the date of index hospitalization to date of death or December 31th, 2010.

### 3. Outcomes and covariate measurements

Baseline comorbidities were identified by ICD-9-CM (International Classification of Diseases, 9th Revision, Clinical Modification) diagnosis codes and medication during index hospitalization. Primary outcomes were major adverse cardiovascular events (MACE) including death, MI and stroke.

### 4. Statistical methods

We used the chi-square test to examine the difference in proportions of categorical variables among the LE, AK and BK groups, and tested continuous variables by one-way ANOVA. Associations between the three groups and complications were identified by multivariate logistic regression analysis. We performed multivariate Cox regressions to reveal the association between the three groups and outcomes (prognoses). In a further subgroup analysis, we created a multivariate Cox model to identify the factors associated with overall mortality for patients receiving major amputations. The results are presented as adjusted odds ratios (OR) for logistic regression, or as adjusted hazard ratios (HR) for Cox regressions, with corresponding 95% confidence intervals (CI). All data analyses were conducted using SPSS software version 15 (SPSS Inc., Chicago, Illinois).

## Results

### 1. Patient characteristics

For our study cohort, we identified a total of 7,588 patients diagnosed with PAD who underwent PTA. Twenty patients under age 18 when undergoing PTA were excluded, resulting in a total of 7,568 adult patients. In the whole cohort there were 4,694 (62%) male patients and 2,874 (38%) female ones. The mean age for the overall cohort was 71.3 years (*SD* = 11.1). The mean follow-up period was 2.45 years (*SD* = 2.54). At the index hospitalization where the patient received PTA, 6,448 patients who remained free of amputation were designated as the LE group, 952 patients who received BK amputation were designated as the BK group, and 168 patients who suffered AK amputation were designated as the AK group.

Table 1 shows the clinical characteristics and comorbidities of the patients. AK patients were older than patients in the two other groups. The prevalence of diabetes, dyslipidemia and end stage renal disease was substantially higher in the BK group compared to the AK group (89% *vs*. 70%; 32% *vs*. 23%; 26% *vs*. 20%, respectively). In contrast, the prevalence of atrial fibrillation was higher in the AK group when compared to the BK group (17% *vs*. 7%).

**Table 1:** Clinical characteristics and comorbidities of the study patients.

		Amputation at the admission			
Characteristics	All patients	LE group	BK group	AK group	*P*
Number of patients	7568	6448	952	168	–
Age, year	71.3±11.1	71.5±11.1	69.8±10.9	73.6±11.4	<0.001
Male sex	4694 (62)	4052 (63)	539 (57)	103 (61)	0.001
Indication					
Type2 DM foot	857 (11)	432 (7)	392 (41)	33 (20)	<0.001
Type1 DM foot	4 (0)	0 (0)	4 (0)	0 (0)	<0.001
Chronic ulceration	513 (7)	398 (6)	108 (11)	7 (4)	<0.001
Gangrene	1279 (17)	689 (11)	529 (56)	61 (36)	<0.001
Comorbidities					
Hypertension	6976 (92)	5927 (92)	884 (93)	165 (98)	0.008
Diabetes	5003 (66)	4040 (63)	845 (89)	118 (70)	<0.001
Dyslipidemia	2998 (40)	2655 (41)	304 (32)	39 (23)	<0.001
Gout	721 (10)	617 (10)	79 (8)	25 (15)	0.026
Stroke	2161 (29)	1830 (28)	277 (29)	54 (32)	0.524
Atrial fibrillation	855 (11)	756 (12)	71 (7)	28 (17)	<0.001
Malignancy	586 (8)	508 (8)	67 (7)	11 (7)	0.559
COPD	828 (11)	705 (11)	102 (11)	21 (13)	0.791
Uremia	1798 (24)	1440 (22)	314 (33)	44 (26)	<0.001
Congestive heart failure	1792 (24)	1501 (23)	242 (25)	49 (29)	0.083
Old myocardial infarction	1003 (13)	872 (14)	110 (12)	21 (13)	0.237
Coronary artery disease	3608 (48)	3194 (50)	349 (37)	65 (39)	<0.001
CABG	552 (7)	478 (7)	60 (6)	14 (8)	0.409
Carotid stenting	103 (1)	98 (2)	4 (0)	1 (1)	0.016
Dialysis	1348 (18)	1072 (17)	243 (26)	33 (20)	<0.001

Abbreviations: COPD = chronic obstructive pulmonary disease; CABG = coronary artery bypass graft; DM = diabetes; LE = Lower extremity preserved; BK = below knee; AK = above knee.

### 2. Events during index hospitalization

Table 2 shows the case number and proportion of various complications for the three groups. The risk of acute renal failure was higher in the AK group than the LE and BK groups (OR = 5.57, 3.90, *P*<0.001). Both the AK and BK groups had a higher risk of newly initiated dialysis compared to the LE group (OR = 1.79, *P* = 0.003, for BK; OR = 3.10, *P* = 0.001, for AK). A significantly higher risk of in-hospital mortality was observed for the AK group when compared to the LE and BK groups (OR = 4.18, 3.28, *P*<0.001). In contrast, these three groups did not differ in the risk of new occurrence of MI or stroke. Results for risk of MACE were similar to the pattern for in-hospital mortality.

**Table 2:** The association of amputation status after PTA with complication at same admission.

	Number of event (%)			Adjusted odds ratio and 95% CI					
Variable	LE(*n* = 6448)	BK(*n* = 952)	AK(*n* = 168)	BK *vs*. LE		AK *vs*. LE		AK *vs.* BK	
				OR (95% CI)	*P*	OR (95% CI)	*P*	OR (95% CI)	*P*
ARF	93 (1.4)	22 (2.3)	14 (8.3)	1.43(0.88–2.32)	0.150	5.57(3.04–10.20)	<0.001	3.90(1.92–7.94)	<0.001
New onset of dialysis	113 (1.8)	38 (4.0)	10 (6.0)	1.79(1.21–2.65)	0.003	3.10(1.56–6.19)	0.001	1.73(0.82–3.63)	0.147
MACE	498 (7.7)	69 (7.2)	25 (14.9)	1.00(0.76–1.32)	0.991	2.11(1.34–3.32)	0.001	2.11(1.26–3.51)	0.004
In-hospital death	177 (2.7)	39 (4.1)	20 (11.9)	1.27(0.88–1.84)	0.195	4.18(2.52–6.94)	<0.001	3.28(1.82–5.90)	<0.001
Myocardial infarction	124 (1.9)	17 (1.8)	4 (2.4)	1.08(0.64–1.84)	0.767	1.33(0.48–3.67)	0.584	1.23(0.40–3.72)	0.718
Stroke	222 (3.4)	19 (2.0)	3 (1.8)	0.79(0.47–1.32)	0.361	0.53(0.16–1.77)	0.302	0.68(0.19–2.44)	0.550

The odds ratio were adjusted for age, gender, diabetes, hypertension, dyslipidemia, previous cerebral vascular accident, heart failure, coronary artery disease, chronic kidney disease, gout, AF, malignancy, chronic obstructive pulmonary disease, center volume and level of hospital.

Abbreviations: ARF = acute renal failure; MACE = major adverse cardiac events; LE = Lower extremity preserved; BK = below knee; AK = above knee; OR = odds ratio; CI = confidence interval.

### 3. Outcomes during Follow-up

Table 3 displays the numbers of cases and proportion of various prognoses one year after PTA and overall for the three groups. No risk of second amputation above the knee or newly initiated dialysis was found for the AK and BK groups. The BK group were at higher risk of suffering a second BK after PTA compared to the LE group (HR = 1.19, *P* = 0.028), whereas the AK group were less likely to suffer BK compared to the LE and BK groups (HR = 0.35, *P* = 0.003; HR = 0.30, *P* = 0.001). One year after index hospitalization with PTA, the risk of death as well as MACE was significantly higher in the AK group than in the LE and BK groups. The BK group had a lower risk of stroke than the LE group (HR = 0.63, *P*<0.05), but there was no statistically significant difference in risk of MI among the three groups.

**Table 3:** The association of amputation status after PTA with prognoses.

	Number of event (%)			Adjusted hazard ratio and 95% CI					
Variable	LE(*n* = 6448)	BK(*n* = 952)	AK(*n* = 168)	BK *vs*. LE		AK *vs*. LE		AK *vs.* BK	
				HR (95% CI)	*P*	HR (95% CI)	*P*	HR (95% CI)	*P*
Above knee amputation	236 (3.7)	48 (5.0)	9 (5.4)	1.23(0.89–1.70)	0.206	1.83(0.94–3.59)	0.077	1.49(0.73–3.06)	0.276
Below knee amputation	945 (14.7)	216 (22.7)	8 (4.8)	1.19(1.02–1.39)	0.028	0.35(0.18–0.71)	0.003	0.30(0.15–0.60)	0.001
New onset of dialysis	179 (2.8)	33 (3.5)	3 (1.8)	1.32(0.89–1.94)	0.162	1.10(0.35–3.48)	0.867	0.84(0.26–2.75)	0.771
MACE (1 year)	1427 (22.1)	239 (25.1)	74 (44.0)	1.00(0.87–1.15)	0.959	1.94(1.53–2.45)	<0.001	1.93(1.49–2.51)	<0.001
Death	1134 (17.6)	218 (22.9)	69 (41.1)	1.14(0.98–1.32)	0.083	2.18(1.70–2.78)	<0.001	1.91(1.45–2.51)	<0.001
Myocardial infarction	122 (1.9)	12 (1.3)	3 (1.8)	0.70(0.38–1.29)	0.253	1.15(0.37–3.65)	0.807	1.64(0.46–5.84)	0.444
Stroke	279 (4.3)	26 (2.7)	4 (2.4)	0.63(0.42–0.95)	0.026	0.61(0.23–1.63)	0.322	0.97(0.34–2.78)	0.948
MACE (Overall)	2983 (46.3)	467 (49.1)	116 (69.0)	1.08(0.98–1.20)	0.128	1.81(1.50–2.18)	<0.001	1.67(1.36–2.06)	<0.001
Death	2529 (39.2)	430 (45.2)	112 (66.7)	1.20(1.08–1.33)	0.001	1.98(1.63–2.40)	<0.001	1.65(1.34–2.04)	<0.001
Myocardial infarction	310 (4.8)	38 (4.0)	8 (4.8)	1.00(0.70–1.41)	0.985	1.49(0.74–3.03)	0.265	1.50(0.70–3.23)	0.301
Stroke	675 (10.5)	69 (7.2)	9 (5.4)	0.83(0.64–1.07)	0.142	0.71(0.37–1.37)	0.304	0.86(0.43–1.72)	0.662

The hazard ratio were adjusted for age, gender, diabetes, hypertension, dyslipidemia, previous cerebral vascular accident, heart failure, coronary artery disease, chronic kidney disease, gout, AF, malignancy, chronic obstructive pulmonary disease, center volume and level of hospital.

Abbreviations: MACE = major adverse cardiac events; LE = Lower extremity preserved; BK = below knee; AK = above knee; HR = hazard ratio; CI = confidence interval.

The AK group had higher risk of overall mortality and MACE than the other two groups. The rate of MACE was similar for the LE and BK groups, but the BK group had a higher risk of death than the LE group (HR = 1.20, *P*<0.01).

### 4. Death causes analysis

Table 4 tabulates the most common causes of death in PAD patients after PTA. For the whole cohort, the leading cause of death was diabetes mellitus (DM) and PAD-related complications (17.2%), followed by cardiovascular disease (15.6%), sepsis (14.3%), pneumonia (11.5%) and respiratory failure (10%), respectively. DM- and PAD-related death is defined as the death due to complications or sequelae induced or aggravated by DM and PAD, including gangrene-related sepsis and complications during PTA or amputation. Patients in the AK group were more likely to expire due to DM and PAD-related complications (30.6%) and less likely to die of respiratory failure (5.1%) and malignancy (0%), when compared to the other groups. DM- and PAD-related complications and sepsis were substantially lower in the LE group (15.9%, 13.4%) compared to the AK and BK groups.

**Table 4:** Cause of mortality (*n* = 2531).

	Overall	LE	BK	AK
Cause of mortality	*n* (%)	*n* (%)	*n* (%)	*n* (%)
Cannot be specified	258 (10.2)	216 (10.4)	36 (10.3)	6 (6.1)
DM and PAD related	435 (17.2)	331 (15.9)	74 (21.1)	30 (30.6)
Sepsis*	362 (14.3)	279 (13.4)	64 (18.3)	19 (19.4)
Pneumonia	290 (11.5)	240 (11.5)	39 (11.1)	11 (11.2)
COPD	20 (0.8)	18 (0.9)	2 (0.6)	0 (0.0)
Cardiovascular disease	395 (15.6)	340 (16.3)	41 (11.7)	14 (14.3)
Stroke	148 (5.8)	127 (6.1)	17 (4.9)	4 (4.1)
Respiratory failure	253 (10.0)	211 (10.1)	37 (10.6)	5 (5.1)
Renal failure	64 (2.5)	53 (2.5)	10 (2.9)	1 (1.0)
GI bleeding and GI system	75 (3.0)	60 (2.9)	11 (3.1)	4 (4.1)
Fall and related mortality	10 (0.4)	7 (0.3)	2 (0.6)	1 (1.0)
Malignancy	162 (6.4)	150 (7.2)	12 (3.4)	0 (0.0)
Others	59 (2.3)	51 (2.4)	5 (1.4)	3 (3.1)

*included UTI, septic septicemia, gangrene, decubitus ulcer.

Abbreviations: DM = diabetes; PAD = peripheral artery disease; COPD = chronic obstructive pulmonary disease; GI = Gastrointestinal; LE = Lower extremity preserved; BK = below knee; AK = above knee.

### 5. Clinical factors associated with death after major amputation of the lower extremity

Table 5 reveals the results of Cox models designed to identify the clinical predictors of overall mortality after BK or AK amputation. Older age (HR = 1.38, *P*<0.001), male gender (HR = 1.19, *P*<0.05), history of heart failure (HR = 1.62, *P*<0.001), history of dialysis (HR = 1.98, *P*<0.001) and receiving PTA in a hospital with high patient volume (HR = 1.23, *P*<0.05) were independently associated with higher risk of death after BK or AK amputation. In the subgroup analysis for patients receiving major amputation at large volume medical centers and low volume centers, the proportion of AK amputations at large volume centers is significantly higher than at low volume centers (19.7% vs. 11.3%, p<0.001). The baseline characteristics, including age and gender, were similar at large volume centers and low volume centers. The comorbidities, including hypertension, diabetes, stroke, malignancy, heart failure, coronary artery disease and myocardial infarction history, were not different between these two groups.

**Table 5:** Factors associated with death after major amputation of the lower extremity (*n* = 1120).

Variable	HR (95% CI)	*P*
Age (per 10 years)	1.38 (1.26–1.51)	<0.001
Male gender	1.19 (1.00–1.42)	0.049
Diabetes	1.13 (0.88–1.44)	0.342
Hypertension	1.10 (0.77–1.58)	0.606
Dyslipidemia	1.06 (0.86–1.29)	0.593
Previous stroke	1.08 (0.89–1.31)	0.428
Heart failure	1.62 (1.32–1.98)	<0.001
Coronary artery disease	0.98 (0.81–1.18)	0.837
Dialysis	1.98 (1.62–2.42)	<0.001
Gout	1.10 (0.82–1.46)	0.535
Atrial fibrillation	1.23 (0.92–1.64)	0.169
Malignancy	1.31 (0.98–1.77)	0.072
Chronic obstructive pulmonary disease	1.04 (0.79–1.37)	0.785
High center volume‡	1.23 (1.02–1.49)	0.029
Medical center	0.92 (0.77–1.11)	0.393

‡ defined as greater than 27 procedures per year.

Abbreviations: HR = hazard ratio; CI = confidence interval.

## Discussion

The previous literature [10] demonstrated that the overall MACE rate was high in the AK and BK groups; however, in our study, the MACE rate was not significantly different for the LE and BK groups. Additionally, the incidence of atrial fibrillation (AF) was higher in the AK group than the two other groups, which may imply different disease mechanisms and treatment strategies for patients undergoing AK amputations.

### 1. Patient characteristics

In our cohort, patients with PAD undergoing PTA were predominantly male, consistent with findings from the previous study [17]. Because risk factors for PAD, such as smoking and hyperlipidemia, are more common among males, the incidence of PAD is higher for them. In addition, male gender was a predisposing factor for mortality in our cohort study. Meanwhile, comorbidities such as hypertension, stroke, coronary artery disease and myocardial infarction had the same prevalence across the three groups, which implied that atherosclerosis may be the common disease mechanism affecting those patients [8]. In coronary artery disease studies, many medications, including those for lipid profile modification [18], blood sugar control [19] or blood pressure control [20], have been used to promote atherosclerotic plaque regression. According to a previous study [21], regression of atherosclerotic coronary plaque volume may represent a surrogate for myocardial infarction and repeat revascularization. Medication that modifies lipid profiles, and those that act on blood sugar metabolism and blood pressure may also induce regression of atherosclerotic plaque in peripheral artery disease, consequently reducing the rate of re-stenosis in peripheral vessels and repeat revascularization.

### 2. Events during index hospitalization and outcomes after discharge

During the index hospitalization, the prevalence of acute renal failure was higher in the AK group than the BK and LE groups. Newly initiated dialysis occurred more frequently in the two amputation groups compared to the LE group (Table 2). However, after long-term follow up, the rate of new dialysis occurring was the same for the three groups (Table 3). So the transient renal function impairment showing in the AK group did not indicate increased risk of dialysis in the long term.

During following up, the frequency of a second amputation, BK, was much higher in the AK and BK groups than the LE group (Table 3). This result reveals that patients undergoing AK or BK amputation at index hospitalization are more likely to have the other leg amputated in the future. However MACE in the BK group was the same as in the LE group.

During one-year follow up, the incidence of stroke was a little lower in the BK group than the LE group, and a slightly higher mortality rate was noted in the BK group compared to the LE group, although the difference was not significant. Higher mortality rates may reduce the incidence of stroke due to the competitive outcome effect. The same scenario was also observed for the AK and LE groups, however the result was not statistically significant (HR: 0.61; 95% CI: 0.23–1.63) because of the relatively small sample size of the AK group.

**Figure 2:**
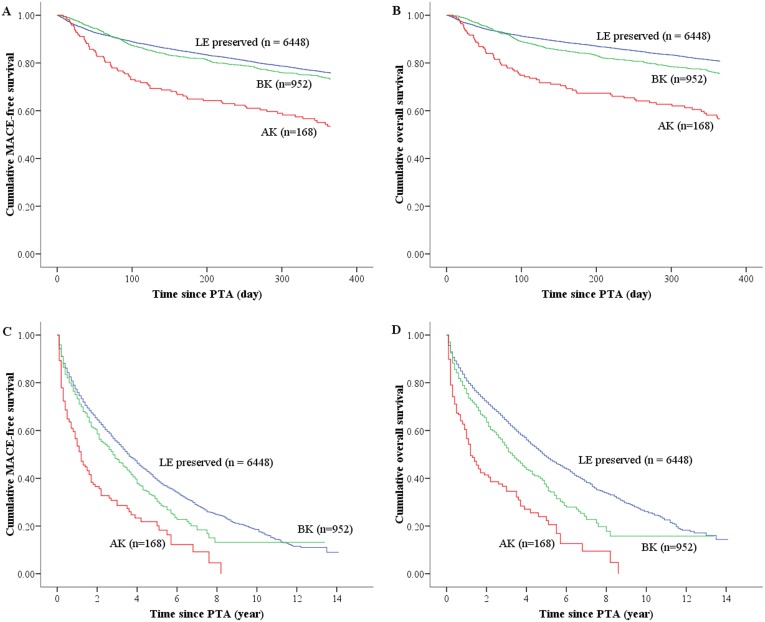
Comparison of prognostics among the study groups. (A) major adverse cardiac events in one year, (B) 1-year mortality, (C) overall major adverse cardiac events, and (D) overall mortality.

Consistent with the previous literatures [9], [10], despite undergoing PTA, high mortality among PAD patients was observed in our study, with or without major amputation. However it was surprising that all-cause mortality showed no statistically significant differences between the LE and BK groups at one-year follow up, but significantly higher mortality was observed in the AK group (Fig. 2B). In long-term follow up, mortality was higher in the BK group than the LE group, but it was highest in AK group (Fig. 2D). However there was no significant difference in MACE between the BK and LE groups in short-term (Fig. 2A) or long-term follow up (Fig. 2C). MACE was unequivocally highest in the AK group.

Although BK amputation, like AK amputation, has been defined as a major amputation [9], [10], [22], the outcomes were very different for those two groups. According to a previous study [23], survival among patients with BK amputations was not better than those with AK amputations, but our results show better outcomes for the BK amputation group and LE group than for the AK group. Our results are compatible with those from the latest study that has been done [10]. Our study shows that avoiding AK amputation should be a key treatment strategy for PAD patients who receive PTA.

### 3. Risk factors associated with outcomes and causes of death

Since avoiding AK amputation is the most important strategy following PTA, it is worth noting some different baseline characteristics of patients in the AK and BK groups. As mentioned previously, AF was more prevalent among the patients in the AK group compared to those in the BK group (17% vs. 7%, p<0.001), which could suggest that arterial embolisms induced by AF may be the cause of some severely ischemic legs needing AK amputation [8], [17], [24], On the other hand, the higher prevalence of DM and dyslipidemia among the patients in the BK group compared to the AK group (89% vs. 70%, p<0.001; 32% vs. 23%, p<0.001) may indicate that atherosclerosis may be a more important cause of PAD in the BK group than in the AK group. So anticoagulation and thrombolysis may be important treatment strategies for avoiding AK amputation in patients who have PAD and AF [22], [25], [26].

Consistent with findings from the existing literature [10], [27], the factors associated with higher mortality included advanced age, heart failure, dialysis, and male gender, all of which could contribute to these extremely high short- and long-term event rates following AK or BK amputation. Unlike other study which revealed better outcomes at medical centers with higher patient volume [28], receiving PTA at high volume medical centers in Taiwan was associated with a higher risk of death after major amputation. According to subgroup analysis for large volume and low volume centers, the proportion of AK amputations in large volume centers is significantly greater than in low volume centers (19.7% vs. 11.3%, p<0.001). The baseline characteristics and comorbidities are similar for the two groups. This means that patients with more severe PAD are sent to large volume centers for peripheral angioplasty, so the proportion of AK amputations is higher in large volume centers, with the result that there seem to be more unfavorable outcomes in large volume centers.

Our study also reviewed the causes of mortality. DM or PAD was the most important cause of death in patients with PAD, even when they received aggressive treatment such as PTA. The risk of sepsis was higher in patients with PAD [29], and was an important cause of mortality. Consistent with previous literature [11], [22], we found that cardiovascular disease is also a major cause of death in patients with PAD. Pneumonia also poses a threat to their lives. In order to reduce mortality among such patients, effective infection prevention and control are very important, in addition to treating the PAD itself. Some PAD patients may suffer walking impairment or even immobility due to lower limb dysfunction. As with stroke patients, immobility can predispose them to pneumonia [30]. Declining ability to walk is also known to affect mortality [31]. Rehabilitation to increase mobility may thus improve both daily activity levels and medical outcomes [32].

## Study Limitations

Our study has a number of limitations. First, the NHI data do not include information about the severity of disease, such as the numbers of diseased vessels and TASC classifications, which may influence both the rate of AK or BK amputation and long-term outcomes following AK or BK amputation. Second, our study cannot distinguish whether new endovascular intervention devices such as stents or drug-eluting balloons, which may affect the prognosis of PAD, were used during PTA. Lastly, our study only suggests trends and possible treatment strategies for improving outcomes among patients with PAD. Randomized controlled trials should be designed to test these different treatment strategies.

## Conclusion

Mortality rates among patients with PAD undergoing PTA remain high. MACE were much higher among patients receiving AK amputation than among those receiving BK amputation or those whose lower extremity were preserved, whether in both long-term or short-term follow up. Short-term and overall MACE were the same for the LE and BK groups. Furthermore, overall mortality increased with increasing area of amputation. Sepsis, cardiovascular disease and pneumonia are the major causes of mortality, in addition to DM and PAD-related complications.
